# Epigenetic reprogramming converts human Wharton’s jelly mesenchymal stem cells into functional cardiomyocytes by differential regulation of Wnt mediators

**DOI:** 10.1186/s13287-017-0638-7

**Published:** 2017-08-14

**Authors:** G. Bhuvanalakshmi, Frank Arfuso, Alan Prem Kumar, Arun Dharmarajan, Sudha Warrier

**Affiliations:** 1 Division of Cancer Stem Cells and Cardiovascular Regeneration, Manipal Institute of Regenerative Medicine, Manipal University, Bangalore, 560 065 India; 2 Stem Cell and Cancer Biology Laboratory, School of Biomedical Sciences, Curtin Health Innovation Research Institute, Curtin University, Perth, WA 6845 Australia; 3 School of Anatomy, Physiology and Human Biology, Faculty of Science, The University of Western Australia, 35 Stirling Highway, Crawley, WA 6009 Australia; 4 Cancer Science Institute of Singapore, National University of Singapore, Singapore, 117599 Singapore; 5 Department of Pharmacology, Yong Loo Lin School of Medicine, National University of Singapore, Singapore, 117597 Singapore; 6 School of Biomedical Sciences, Curtin Health Innovation Research Institute, Curtin University, Perth, WA 6102 Australia; 7 National University Cancer Institute, Singapore, 119074 Singapore; 8 Department of Biological Sciences, University of North Texas, Denton, TX 76203-5017 USA; 9 Curtin Medical School, Faculty of Health Sciences, Curtin University, Perth, WA 6875 Australia

**Keywords:** Mesenchymal stem cells, Epigenetic modification, Cardiomyocytes, DNA methyltransferase, Histone deacetylase, Wharton’s jelly

## Abstract

**Background:**

Lineage commitment of mesenchymal stem cells (MSCs) to cardiac differentiation is controlled by transcription factors that are regulated by epigenetic events, mainly histone deacetylation and promoter DNA methylation. Here, we studied the differentiation of human Wharton’s jelly MSCs (WJMSCs) into the cardiomyocyte lineage via epigenetic manipulations.

**Methods:**

We introduced these changes using inhibitors of DNA methyl transferase and histone deacetylase, DC301, DC302, and DC303, in various combinations. We characterized for cardiogenic differentiation by assessing the expression of cardiac-specific markers by immunolocalization, quantitative RT-PCR, and flow cytometry. Cardiac functional studies were performed by FURA2AM staining and Greiss assay. The role of Wnt signaling during cardiac differentiation was analyzed by quantitative RT-PCR. In-vivo studies were performed in a doxorubicin-induced cardiotoxic mouse model by injecting cardiac progenitor cells. Promoter methylation status of the cardiac transcription factor Nkx2.5 and the Wnt antagonist, secreted frizzled-related protein 4 (sFRP4), after cardiac differentiation was studied by bisulfite sequencing.

**Results:**

By induction with DC301 and DC302, WJMSCs differentiated into cardiomyocyte-like structures with an upregulation of Wnt antagonists, sFRP3 and sFRP4, and Dickkopf (Dkk)1 and Dkk3. The cardiac function enhancer, vinculin, and DDX20, a DEAD-box RNA helicase, were also upregulated in differentiated cardiomyocytes. Additionally, bisulfite sequencing revealed, for the first time in cardiogenesis, that sFRP4 is activated by promoter CpG island demethylation. In vivo, these MSC-derived cardiac progenitors could not only successfully engraft to the site of cardiac injury in mice with doxorubicin-induced cardiac injury, but also form functional cardiomyocytes and restore cardiac function.

**Conclusion:**

The present study unveils a link between Wnt inhibition and epigenetic modification to initiate cardiac differentiation, which could enhance the efficacy of stem cell therapy for ischemic heart disorders.

**Electronic supplementary material:**

The online version of this article (doi:10.1186/s13287-017-0638-7) contains supplementary material, which is available to authorized users.

## Background

Cardiac development is exquisitely regulated by a plethora of transcription factors, and its complexity is reflected by the fact that mutations in these factors during development can cause congenital heart diseases at birth. Despite advances in the prevention and management of cardiovascular diseases, this multifactorial disease remains the leading global cause of death, accounting for 17.3 million deaths per year, and is expected to grow to more than 23.6 million by 2030 [1]. As the regenerative potential of cardiac tissue is limited in adult life, leading to degeneration in pathological conditions such as myocardial infarction, stem cell-based therapy has been increasingly used to improve outcome in cardiac disease. One of the most promising advances in stem cell therapy for cardiovascular diseases has been the allogeneic transplantation of immunocompromised mesenchymal stem cells (MSCs) into patients, which is now in an immune-privileged clinical trial [2]. MSCs from various fetal and adult tissues are excellent viable options for stem cell therapy for cardiac degeneration.

Commitment to the cardiac lineage is initiated by specific signals that regulate a set of transcription factors which program cardiogenesis. After the generation of cardiomyocytes, there is proliferation throughout development so that the fetal heart increases in size. Soon after birth, there is limited proliferation, with the cells undergoing terminal differentiation and eventually permanently exiting from the cell cycle [3]. During myocardial damage and injury, cardiac progenitor cells migrate to the site of injury to augment the limited mitotic capacity of terminally differentiated cardiomyocytes and eventually regenerate the myocardium [4]. However, these cell populations are very few in number and decrease significantly during the aging process, thereby compromising the regenerative process [5]. Pluripotent, embryonic and mesenchymal stem cells, by virtue of their ability to self-renew and differentiate into multiple lineages, are emerging as viable alternatives to replace the lost cardiomyocytes [6, 7]. MSCs from adult tissues differ from MSCs from tissues of fetal origin by way of their proliferative ability, immunosuppressive properties, and therapeutic efficacy; the use of Wharton’s jelly, the primitive connective tissue of the human umbilical cord, is a viable alternate perinatal source of MSCs [8, 9].

Induction of the mesoderm to the cardiovascular cell lineages is coordinated by the effectors of the Wnt/β-catenin signaling pathway [10] and transforming growth factor-beta superfamily [11]. The role of epigenetic remodeling in cardiac differentiation has been revealed by mutations in chromatin modifiers such as ATP-dependent chromatin remodeling complex (CHD7) [12], Williams syndrome transcription factor (WSTF) [13], and histone H3K36 methyltransferase Wolf-Hirschhorn syndrome candidate 1 (WHSC1) [14]—all of which are also involved in the pathogenesis of human heart diseases [15]. Although several epigenetic modifiers have been used in cardiac differentiation, the differentiation efficacy of the clinically relevant MSCs to cardiomyocytes remains poor. Furthermore, little is known about how Wnt mediators and antagonists are regulated by epigenetic modifications to drive cardiomyogenesis.

In this study, we investigate the effect of inhibitors of two main epigenetic processes, DNA methylation and histone deacetylation, on cardiomyogenesis from Wharton’s jelly MSCs (WJMSCs), through functional cardiac assays and in-vivo engraftment of derived cardiomyocytes, and reveal an epigenetic remodeling of Wnt antagonists during cardiac differentiation.

## Methods

### Isolation and expansion of WJMSCS

All study procedures were approved by the Institutional Ethical Committee (IEC) of Manipal Hospital, Bangalore, India. After obtaining informed consent, fresh human umbilical cords (*n* = 20) were obtained and stored in Dulbecco’s phosphate-buffered saline (DPBS; Invitrogen, Carlsbad, CA, USA), containing antibacterial and antimycotic agents, prior to tissue processing. Isolation of WJMSCs by enzymatic digestion was performed according to the method of Wang et al. [16] with some modifications. The cord was cut into pieces of 4–5 cm and, after removal of blood vessels, the Wharton’s jelly was scraped off and was washed three to five times with sterile DPBS. Next, the tissue was subjected to enzymatic digestion with 0.2 g/ml of collagenase (Sigma-Aldrich, St. Louis, MO, USA) for 16 h at 37 °C. The digestion was neutralized by adding equal volumes of medium with 10% fetal bovine serum (FBS; Hyclone, USA), filtered through a 100-μm cell strainer (BD Biosciences), and pelleted at 1500 rpm for 10 min. Finally, cells were washed and cultured in growth medium (DMEM-HG) supplemented with penicillin (100 U/ml), streptomycin (100 μg/ml), and 10% FBS, and incubated at 37 °C with 5% CO_2_. Cells were first passaged, after 8–10 days, until the formation of colony-forming units and then after every 3–4 days. When confluency was achieved, cells were recovered using 0.25% Trypsin–EDTA and passaged further. WJMSCs at passage 3 were used for flow cytometric analysis, differentiation, and further analysis in this study.

### Characterization of WJMSCs

At the third passage, WJMSCs were characterized for bona-fide MSC markers by flow cytometry, RT-PCR, and immunocytochemistry

### Trilineage differentiation

At the third passage, WJMSCs were induced to differentiate into the defining tri-lineages of MSCs; namely osteogenic, adipogenic, and chondrogenic lineages using the protocols that we have previously reported [17, 18].

### Differentiation of WJMSCS into cardiogenic lineage

WJMSCs were grown to 100% confluence on 0.2% gelatin coated plates and then subjected to individual or a cocktail of proprietary inhibitors, DC301 (8 μM, a DNA methyltransferase 1 (DNMT1) specific inhibitor), DC302 (300 nM) and DC303 (0.5 μM), both of which are specific histone deacetylase 1 (HDAC1) inhibitors, and various combinations of these inhibitors (DC301 + DC302, DC301 + DC303, and DC302 + DC303)—for 48 h in DMEM-HG growth medium containing 5% FBS. After 48 h, the induction medium was replenished and incubated for a further 24 h, after which the induction medium was replaced with DMEM-HG growth medium containing 5% FBS, and maintained at 37 °C with 5% CO_2_ and 95% air for up to 2 weeks. Cardiac progenitors (CP) obtained within 5–7 days and differentiated cardiomyocytes obtained within 10–12 days were used for further experiments.

### Immunocytochemistry

WJMSCs from passage 3 or differentiated cardiomyocytes were grown in chamber slides and fixed using 4% paraformaldehyde for 20 min at 4 °C, and were blocked with 3% bovine serum albumin (BSA) in PBS for 30 min at room temperature. The cells were then incubated for 1 h in dark conditions at 4 °C with primary nonlabeled mouse anti-human antibodies against vimentin (1:500 dilution), GATA4 (1:500 dilution), cardiac actin (1:500 dilution), troponin I (TnI) (1:500 dilution), troponin T (TnT) (1:500 dilution), desmin (1:500 dilution), and atrial natriuretic peptide (ANP) (1:500 dilution; all antibodies purchased from BD Biosciences, San Diego, CA, USA) followed by anti-mouse goat fluorescein isothiocyanate (FITC)-labeled secondary antibody (1:1000 dilution; Invitrogen) or anti-rabbit goat phycoerythrin (PE)-labeled secondary antibody (1:1000 dilution; Invitrogen) for 1 h at 37 °C. An additional step was included for staining the intracellular, nuclear, or cytoskeletal markers (vimentin, cardiac actin, TnI, TnT, desmin, and ANP) by washing the cells with PBS containing 0.05% Tween-20, along with the treatment of cells using 3% BSA containing 0.1% Triton X-100 for 30 min at room temperature to ensure that cell permeability was achieved. Nuclei were counter-stained with 4′,6-diamidoino-2-phenylindole (DAPI) (1:10,000 dilution) for 45 sec, and a drop of anti-fade (Vectashield; Vector Laboratory, Burlingame CA, USA) was added to avoid quenching of the fluorochrome. The slides were examined under a Nikon Eclipse TE2000-U fluorescent microscope and images were taken using Qimaging QICAM-fast 1394 (Surrey, BC, Canada).

### Flow cytometry

WJMSCs from passage 3 or differentiated cardiomyocytes were washed with PBS-Tween buffer (PBST) and fixed in prechilled 70% ethanol. WJMSCs were incubated in mouse anti-human FITC-labeled antibodies against CD73, CD90, CD105, or CD34 (1:100 dilution; all antibodies purchased from Becton Dickinson, San Diego, CA, USA) for 1 h on ice. Differentiated cardiomyocytes were incubated with unlabeled primary anti-human mouse troponin I antibody (1:100 dilution; BD Biosciences) for 1 h on ice. After washing with PBST buffer, the differentiated cardiomyocytes were incubated for 30 min with anti-mouse rabbit FITC-labeled secondary antibodies (Invitrogen). The cells were acquired using a BD-FACS Calibur flow cytometer with a 488-nm laser, and data were analyzed by Cell Quest Software (Becton Dickinson, San Jose, CA, USA).

### Semi-quantitative reverse-transcription PCR

Total RNA was extracted from untreated WJMSCs and WJMSCs treated with epigenetic modifiers, using the RNeasy Plus Mini kit (Queen) according to the manufacturer’s instructions. One microgram of total RNA was reverse-transcribed using the SuperScript III First-Strand Synthesis System (Invitrogen). One microgram of total RNA was mixed with 1 μl Oligo dT (50 μM) and 1 μl of dNTP (10 mM), and made up to 13 μl with DEPC-treated water and heated at 65 °C for 10 min, followed by incubation on ice. After primer hybridization, a 7 μl reaction volume containing 5× first-strand buffer, RNase OUT (40 U/μl), 0.1 M DTT, and Superscript III was added to the RNA and subjected to thermocycling (25 °C, 5 min; 50 °C, 60 min; 70 °C, 15 min) in a Veriti 96-well thermal cycler (Applied Biosystems). Qualitative expression of cardiac-specific markers—*GATA4*, *Nkx2.5*, *myosin light chain* (*MLC*), *TnT*, *cardiac actin*, *ANP*, *sFRP1–5*, *Dkk 1* and *3*, *CreB*, *calcineurin* (*CalN*), *vinculin* (*Vcl*), *DDX20*, and *glyceraldehyde 3-phosphate dehydrogenase* (*GAPDH*) (primers were from Sigma, sequence as indicated in Additional file 1: Table S1, S2)—were analyzed by PCR (95 °C, 30 s; annealing temperature, 30 s; 72 °C, 30 s for 40 cycles) in a Veriti 96-well thermal cycler. Products were separated by 1.5% agarose gel electrophoresis and detected using ethidium bromide. Integrated density values (IDV) were calculated using Alpha Manager (San Jose, CA, USA).

### Real-time quantitative reverse-transcription PCR

The qualitative results of mRNA expression were further quantified using iQ SYBR Green Supermix (Bio-Rad, Hercules, CA, USA) in a real-time PCR system. cDNAs and gene-specific primers were mixed with 2× iQ SYBR Green Supermix (Bio-Rad), and dispensed on a MicroAmp® Optical 8*-*Tube Strip. Fluorescence shift was observed using a 7500 Real-time PCR system (Applied Biosystems, Carlsbad, CA, USA). Reaction parameters were 50 °C for 2 min, 95 °C for 10 min, followed by 40 cycles of 95 °C for 15 s and 60 °C for 1 min. The genes *Sox2*, *Oct4*, *CD44*, *CD34*, and *Nanog* were analyzed from cDNA obtained from WJMSCs for stemness and pluripotency traits. WJMSCs treated with epigenetic modifiers were analyzed for cardiac-specific genes *cardiac actin*, *GATA4*, *Nkx2.5*, *MLC*, *TnT*, and *ANP*, and for noncardiac markers such as *PPAR*γ for adipocytes, *collagen II* for chondrocytes, and *osterix* for osteocytes. Wnt-related and other genes that were studied were *sFRP1–5*, *Dkk 1 and 3*, *CreB*, *CalN*, *Vcl*, and *DDX20* (primers are from Sigma Aldrich, sequence as indicated in Additional file 1: Table S1, S2, and S3). The relative abundance of mRNAs was obtained using the comparative cycle threshold method and was normalized to the housekeeping control *GAPDH*. Results were also expressed as fold changes in the mRNA levels of a gene compared to the treated or untreated samples.

### Determination of intracellular calcium release

The increase in intracellular calcium levels after cardiac differentiation of WJMSCs was determined using the fluorescent radiometric Ca^2+^ indicator Fura-2 acetoxymethyl ester (Fura-2, 1 μmol/L; Molecular Probes, Invitrogen, Carlsbad, CA, USA) as reported previously [19]. After induction of WJMSCs with DC301 + DC302 as described, the cells were washed and Fura-2 (1 μmol/L; Molecular Probes) was added to the cells in plain medium and incubated for 37 °C for 45 min. Fluorescent intracellular Ca^2+^ flux was identified by fluorescence microscopy (450–480 nm) and calorimetrically at 480 nm.

### Nitric oxide release assay (Greiss assay)

A modified Griess reagent kit (Sigma Aldrich) was used to detect nitric oxide (NO) release after cardiac differentiation. After WJMSCs were induced to differentiate with various combinations of epigenetic modifiers as described previously, an equal number of cells were plated in 96-well plates with a density of 10,000 cells/well. After 24 h, 100 μl of Griess reagent was added to each well and incubated for 15–20 min in a dark room, and absorbance was measured at 540 nm using a Victor 3 Multilabel Plate Reader (Perkin-Elmer, Waltham, MA, USA).

### Bisulfite modifications and methyl-specific PCR

Genomic DNA was extracted from WJMSCs and differentiated cardiomyocytes using the phenol-chloroform extraction method. To identify the CpG island methylation status, the DNA was subjected to bisulfite modification using the EpiTect plus DNA bisulfite kit (Qiagen, Venlo, the Netherlands) following the manufacturer’s instructions. Methyl and unmethyl specific primers (Additional file 1: Table S4) for *Nkx 2.5* and *sFRP4* were designed using MethPrimer (The Li Lab Software). Methyl-specific PCR was performed as follows: 95 °C for 3 min, 40 cycles of 95 °C for 30 s, 55 °C for 1 min, 72 °C for 1 min, and 72 °C for 7 min. The product was loaded on a 2% agarose gel and bands observed under an UV illuminator and imaged using Alpha Imager (San Jose, CA, USA).

### Bisulfite sequencing

Bisulfite-converted DNA from WJMSCs and differentiated cardiomyocytes was amplified using bisulfite-specific primers for the promoter regions of *Nkx2.5* and *sFRP4*. The amplicons were then scaled up by bulk PCR, and the eluted products (extracted using the QIAquick gel extraction kit; Qiagen) were sequenced by Sanger’s sequencing following a standard protocol [20]. The results comparing the methylation status were analyzed using BiQ Analyzer, a software tool for DNA methylation analysis.

### In-vivo model of cardiac injury and cellular transplantation

In order to study the effect of untreated and treated WJMSCs in a cardiac injury model, cardiac damage was induced in male C57BL/6 mice following the protocol of Desai et al. [21] with some modifications. All animal experiments were performed at Anthem Biosciences Ltd (Bangalore, India) after approval by the Institutional Animal Ethics Committee. Male C57BL/6 mice (25–30 g body weight, *n* = 4) aged 10–12 weeks were used to create an in-vivo model for cardiac damage, induced by intraperitoneal injection of 2.5 mg doxorubicin (dox)/kg body weight, thrice weekly for 2 weeks. Control animals (*n* = 4) were injected intraperitoneally with normal saline (5 ml/kg body weight). Cardiac fibrosis was assessed by staining sections of the heart with hematoxylin and eosin (H&E) and Masson’s trichrome.

After detection of cardiac damage by dox, the mice were injected with either untreated WJMSCs or with CP, which were ascertained to be positive for troponin I by flow cytometry. Control animals received DMEM medium on day 0, after dox treatment. WJMSCs or CP (1 × 10^6^ cells of each type/animal, *n* = 4) were administered as a single dose intravenously on day 0, after dox treatment. Prior to injection, the cells (WJMSCs and CP) were fluorescently labeled with CellTracker™ probe (1:1000 dilution; Invitrogen) by incubating for 15 min and washing with medium. The body weight was measured every alternate day. Animals were sacrificed after 18 days by CO_2_ asphyxiation. The heart was excised, observed for necrosis, and then either fixed in 10% formalin for sectioning or stored in TRIZOL for gene analysis.

### Histological analysis

After the animals were sacrificed, sections of the heart (5 μM) were generated using standard histological techniques. Paraffin sections were prepared from Bouin’s fixed samples using standard procedures [22]. H&E and Masson’s trichrome staining were all performed according to the manufacturer’s instructions (Sigma Diagnostics, St Louis, MO, USA). Slides were examined under a brightfield phase-contrast microscope (Nikon-Eclipse TE 2000-S) and images were taken using Qimaging QICAM-fast 1394 (Surrey, BC, Canada). Fibrotic areas and tissue architecture were analyzed in H&E sections, and fibrosis and collagen content was examined using Masson’s trichrome staining.

### Statistical analysis

All data were represented as mean and SE obtained from experiments performed in triplicate. Statistical significance was assessed by the Student’s unpaired *t* test. For all statistical analyses, *p* <0.05 was considered significant.

## Results

### Isolation and characterization of WJMSCs

After isolating WJMSCs, we first characterized them for MSC-like properties, as shown in Additional file 2: Figure S1. First, we observed cells from colony-forming units forming a homogeneous mat of cells (Additional file 2: Figure S1A1), which were positive for the typical MSC marker, vimentin, by immunohistochemistry (Additional file 2: Figure S1A2). We then characterized for the gene expression of pluripotency markers such as *Sox2*, *Oct4*, and *Nanog*, and for the MSC positive CD marker CD44 and negative marker CD34, and found that *Oct 4*, *Nanog*, and CD44 were highly expressed whereas CD34 expression was minimal (Additional file 2: Figure S1A3). Flow cytometric analyses of WJMSCs revealed the presence of characteristic CD markers, CD73 (90.84%), CD90 (90.91%), and CD105 (88.77%), and only 3% were CD34 positive (Additional file 2: Figure S1B). Final confirmation of the MSC-like phenotype of this cell population was achieved by their ability to differentiate into the trimesodermal lineages, namely adipocytes (Additional file 2: Figure S1C1), osteocytes (Additional file 2: Figure S1C2), and chondrocytes (Additional file 2: Figure S1C3).

### Differentiation of WJMSCs with various epigenetic modifiers

**Fig. 1:**
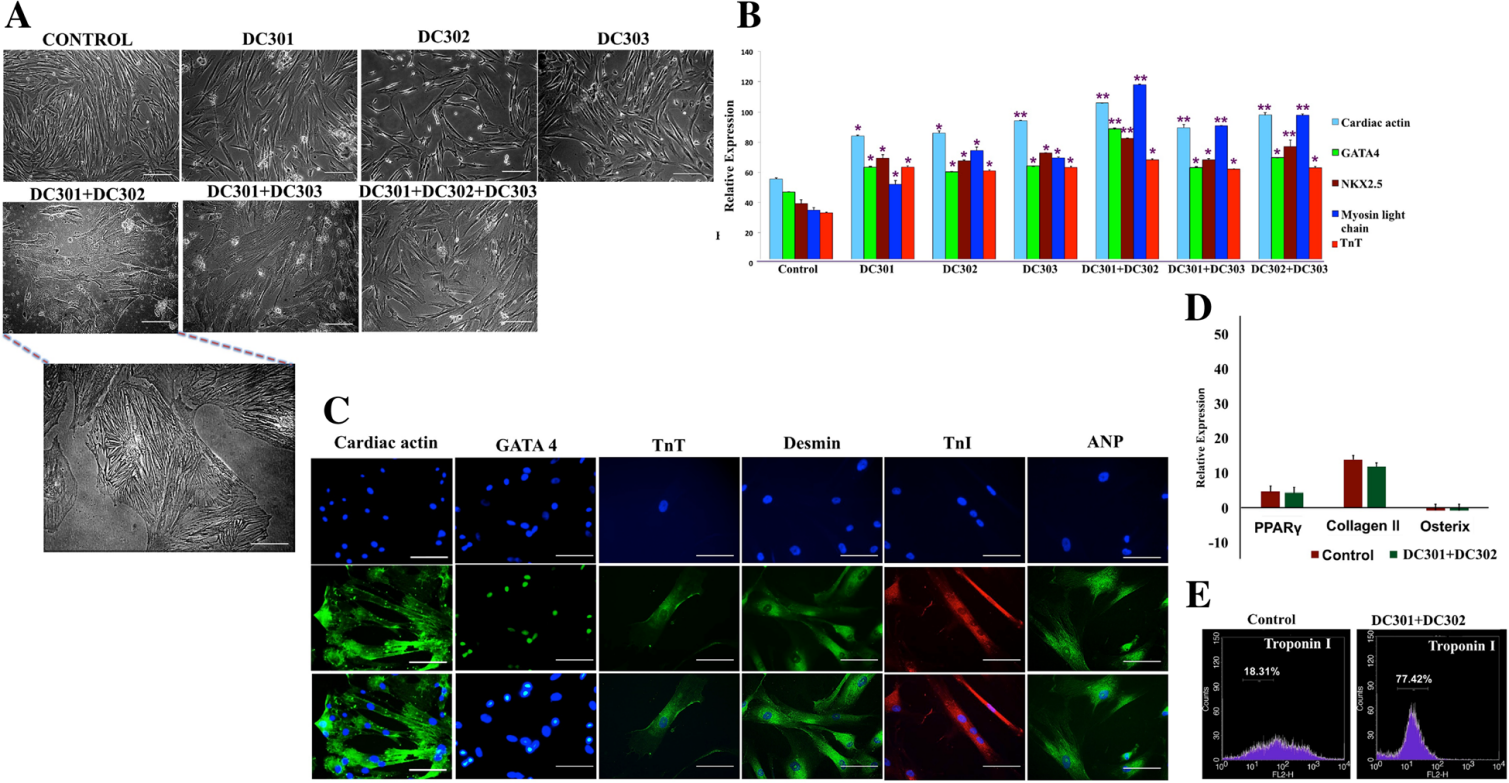
**a** Study of cardiac morphology after induction with different epigenetic modifiers. Differentiation was performed with DC301, DC302, and DC303, and various combinations of these inhibitors, and the appearance of cardiac morphology was assessed by phase-contrast microscopy (*scale bars* = 100 μm, *n* = 3). Treatment with DC301 + DC302 indicates clear cardiac morphology (*scale bar* = 50 μm). **b** Analysis of cardiac markers by quantitative RT-PCR after cardiac induction with different epigenetic modifiers. Cardiac-specific genes *GATA4*, *Nkx2.5*, *MLC*, *TnT*, and *cardiac actin* were studied for their expression after cardiac induction with DC301, DC302, and DC303, and various combinations of these inhibitors as indicated. Results are mean ± SD of three independent experiments performed in triplicate (*p < 0.05, **p < 0.01, *n* = 3). **c** Structural markers of cardiomyocytes identified by immunocytochemistry using antibodies against cardiac actin (*scale bars* = 100 μm), GATA4, TnT, desmin, and troponin I, and visualized using fluorescence microscopy (*scale bars* = 50 μm, *n* = 3). **d** Markers of noncardiac lineages studied by qRT-PCR, which showed no change in osterix, PPARγ, and collagen II levels after treatment of WJMSCs with DC301 + DC302. **e** Flow cytometric analysis performed to quantify TnI-positive cells in WJMSC-derived cardiomyocytes. *ANP* atrial natriuretic peptide, *TnI* troponin I, *TnT* troponin T, *WJMSC* Wharton’s jelly mesenchymal stem cell

After isolation and characterization of human WJMSCs from the umbilical cord (Additional file 2: Figure S1), we subjected WJMSCs (passage 3) to various epigenetic modifiers; namely the specific DNMT1 inhibitor DC301, the HDAC1 inhibitors DC302 and DC303, and various combinations of these markers. After induction for 24 h and incubation for 9 days, we observed differentiation with a change in morphology into a cardiomyocyte phenotype, seen best in the MSCs treated with DC301 and DC302 (Fig. 1a). We next analyzed the expression of early (*Nkx2.5*, *GATA4*) and late (*MLC*, *TnT*, *cardiac actin*) cardiac markers in MSCs differentiated by various combinations of epigenetic modifiers using quantitative RT-PCR (Fig. 1b). We observed that, although there was an increase in the expression of cardiac genes in all of the conditions tested, the expression was much higher in MSCs treated with DC301 and DC302. Therefore, we used MSCs treated with DC301 + DC302 for most of the further downstream studies.

### Functional characterization of MSC-derived cardiomyocytes

To confirm the identity of these differentiated cardiomyocytes, we analyzed the cells for characteristic functional cardiac proteins. Using immunocytochemistry, cardiac actin, TnI, TnT, desmin, and ANP were seen to localize as cytoplasmic striations. GATA4, an early transcription factor, was seen to localize in the nuclear region (Fig. 1c). In addition, three noncardiac lineages (ostoegenic, chondrogenic, and adipogenic) and their respective specific markers, osterix, collagen II, and PPARγ, were examined after treatment of WJMSCs with DC301 + DC302. We observed lack of expression of these specific markers, confirming that DC301 + DC302 (Fig. 1d) treatment did not promote differentiation into these lineages. Next, we analyzed the total population of differentiated cardiomyocytes using the differentiated cardiomycyte marker TnI by flow cytometry. It was observed that 77% of the population was positive for TnI, indicating the efficiency of cardiomyocyte differentiation after treatment of WJMSCs with DC301 + DC302 (Fig. 1e).

### Analysis of Wnt antagonists in MSC-derived cardiomyocytes revealed upregulation of sFRP4 and Dkk1 and Dkk3

**Fig. 2:**
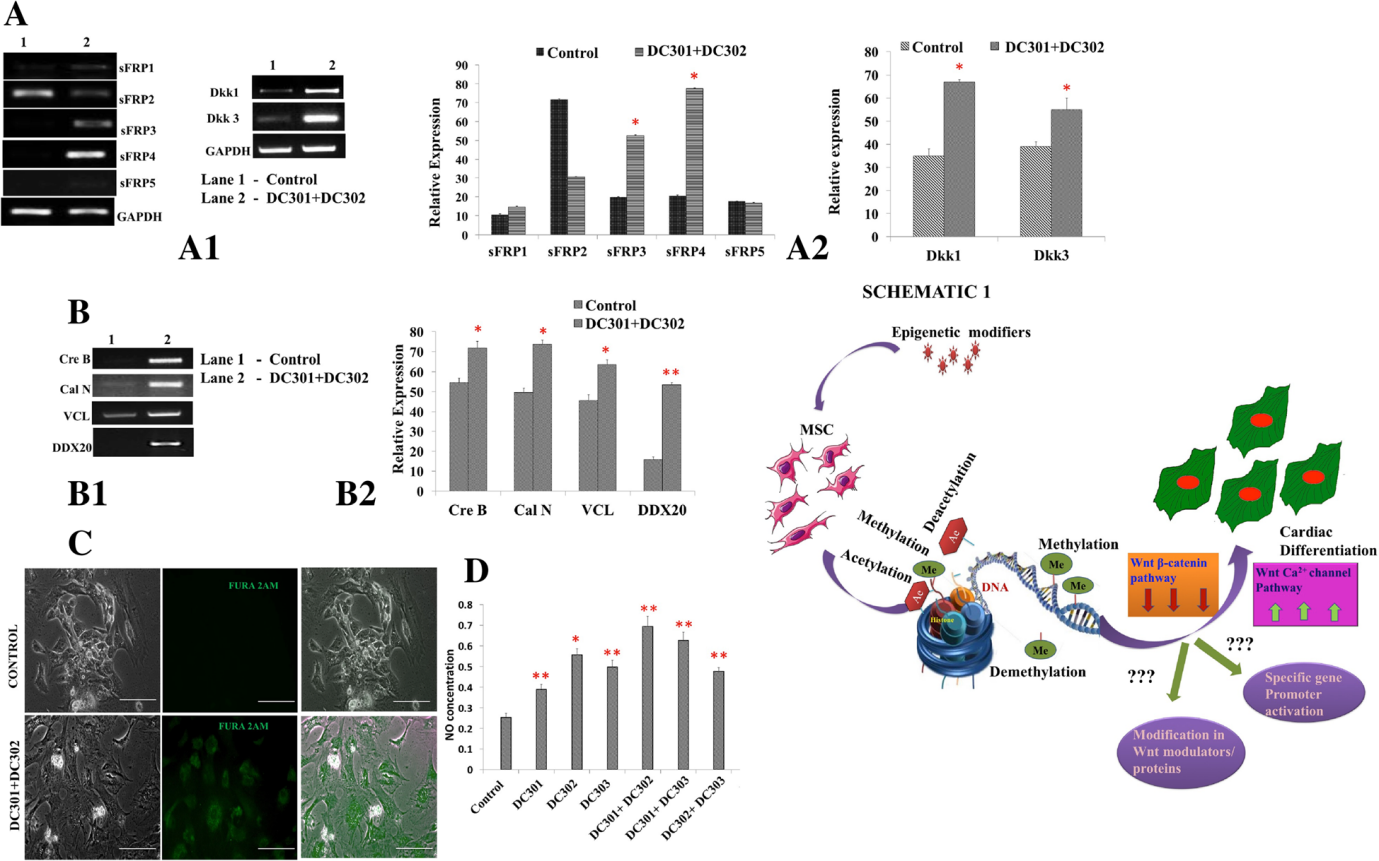
Molecular analysis of Wnt antagonism and related mechanisms in WJMSC-derived cardiomyocytes. **a, b** Wnt antagonists (sFRP1–5, Dkk 1 and 3) and Wnt-related genes (*CreB*, *calcineurin* (*CalN*), *vinculin* (*VCL*), and *DDX20*) were analyzed by semi-quantitative (*A1*, *B1*) and quantitative (*A2*, *B2*) RT-PCR for untreated and DC301 + DC302-treated WJMSCs (*p < 0.05, **p < 0.01, *n* = 3). **c** Calcium release for control and DC301 + DC302-treated WJMSCs was monitored by FURA2M staining (*scale bars* = 100 μm, *n* = 3). **d** Release of nitric oxide (*NO*) accompanying cardiac differentiation was studied by Griess assay for WJMSCs induced with DC301, DC302, and DC303, and various combinations of these inhibitors as indicated (*p < 0.05, **p < 0.01, *n* = 3). *Schematic 1*: representation of the development of cardiomyocytes from MSCs by epigenetic modification via Wnt mediators. *Dkk* Dickkopf, *sFRP* secreted frizzled-related protein, *MSC* mesenchymal stem cell

Based on reports of Wnt antagonism in cardiac differentiation [23, 24], we analyzed the expression of Wnt antagonists of the secreted frizzled-related protein (sFRP) family, sFRP1–5, and the Dickkopf (Dkk) family, Dkk 1 and 3. Although sFRP1, sFRP2, and sFRP4 have been implicated in cardiomyogenesis and ischemic repair [25–27], the expression profile of the sFRP family during cardiac differentiation from MSCs has not been studied. We found that among *sFRP1–5*, expression of *sFRP4* was the most prominent during cardiac differentiation from WJMSCs (Fig. 2aA1). There was also a concomitant increase in the expression of *Dkk1* and *Dkk3* (Fig. 2aA2).

### Wnt-related genes and structural genes were upregulated during cardiomyogenesis

*CreB* and *CalN*, which belong to the Wnt/Ca^2+^ signaling pathway, were examined for their mRNA expression levels, and we found that both were expressed more in the treated cells than in the undifferentiated control. We also analyzed the expression of *VCL*, a Wnt-related structural gene that has been implicated in preserving cardiomyocytes from aging [28]. *Vinculin* expression was higher in differentiated cardiomyocytes than in undifferentiated control MSCs (Fig. 2bB1, B2). *DDX20*, an RNA helicase, was another gene that was analyzed. DDX20, or DEAD-box protein 103, which is essential for early embryonic development, has been known to indirectly interact with GATA4 [29]. On examination of the expression of *DDX20*, we found it to be highly overexpressed in differentiated cardiomyocytes (Fig. 2bB1, B2).

### Calcium release was observed in MSC-derived cardiomyocytes

Intracellular Ca^2+^ transients regulating excitation and contraction are an important event accompanying successful differentiation into cardiomyocytes [30]. Using a calcium-sensitive dye, FURA2M, we observed clear intracellular localization of Ca^2+^ in only differentiated cardiomyocytes and not in the untreated MSCs. There was a prominent cytoplasmic localization of Ca^2+^, suggesting that the cardiomyocytes may be primed for contraction (Fig. 2c).

### Cardiomyogenesis is accompanied by nitric oxide release

NO is an important physiological messenger of cardiac differentiation from embryonic stem cells [31]. NO is a diffusible free radical and signaling molecule, which has an important role in controlling the heart rate, contractibility, and cardiac development [32]. However, no study has so far ascertained NO levels in cardiomyocytes differentiated from human MSCs. We used the classic Griess reagent for measuring levels of NO in MSCs differentiated to cardiomyocytes using various cocktails. The highest accumulation of NO (twofold over the control) was observed in cardiomyocytes derived from DC301 + DC302 treatment (Fig. 2d). These results confirmed the functionality of the differentiated cardiomyocytes.

### Cardiomyogenesis results in promoter demethylation of Nkx2.5 and sFRP4

**Fig. 3:**
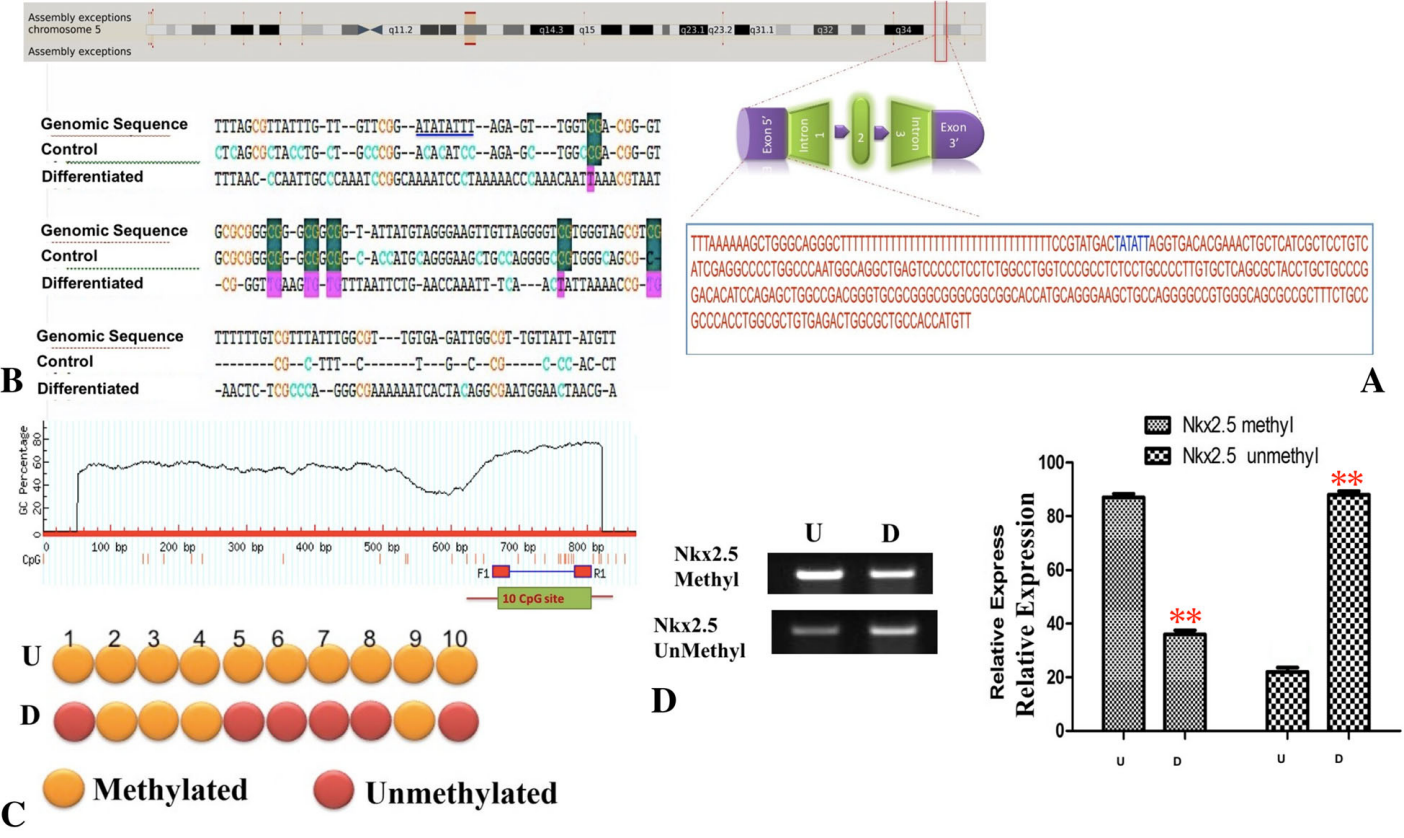
*Nkx2.5* promoter CpG island demethylation. **a** Position of *Nkx2.5* gene on chromosome 5, sequence of the promoter region indicated. **b** After bisulfite conversion, the 160-bp promoter region of *Nkx2.5* was amplified and cloned for bisulfite sequencing for control (*U*) and differentiated cardiomyocytes (*D*); *shaded regions* indicate the CpG islands that are modified after cardiac differentiation. **c** CpG islands were evaluated as methylated (*orange dot*) and unmethylated (*red dot*) sites by bisulfite sequencing. **d** After bisulfite conversion, methylation-specific PCR was performed for *Nkx2.5* using unmethylated and methylated primers in control (U) and differentiated cardiomyocytes (D) (**p < 0.01, *n* = 3, all experiments performed in triplicate) (Color figure online)

**Fig. 4:**
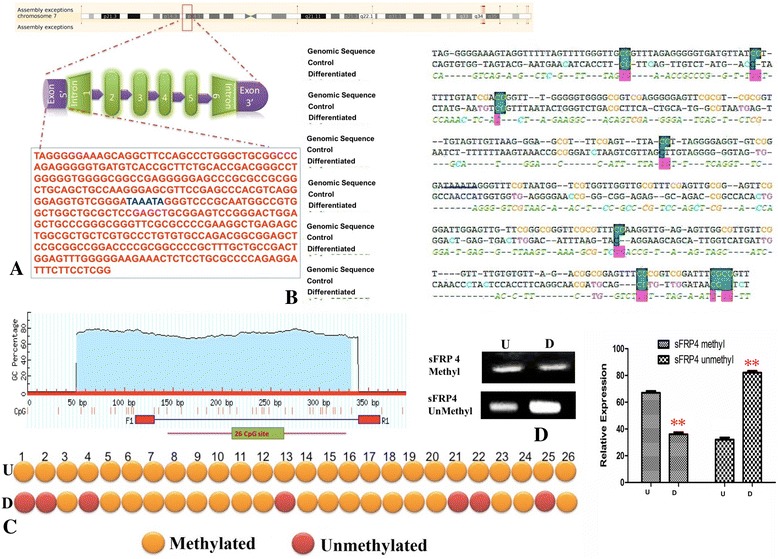
Promoter demethylation patterns of *sFRP4* after cardiomyogenesis. **a** Position of sFRP4 gene on chromosome 7, sequence of the promoter region indicated. **b** After bisulfite conversion, the 254-bp promoter region of *sFRP4* was amplified and cloned for bisulfite sequencing for control (*U*) and differentiated cardiomyocytes (*D*); *shaded regions* indicate the CpG islands that are modified after cardiac differentiation. **c** CpG islands were evaluated as methylated (*orange dot*) and unmethylated (*red dot*) sites by bisulfite sequencing. **d** After bisulfite conversion, methylation-specific PCR was performed for *sFRP4* using unmethylated and methylated primers in control (U) and differentiated cardiomyocytes (D) (**p < 0.01, *n* = 3, all experiments performed in triplicate). *sFRP* secreted frizzled-related protein (Color figure online)

To understand molecular changes in the gene structure accompanying cardiogenesis, we investigated the epigenetic changes in the promoter regions of the early cardiac gene, *Nkx2.5*. To delve deeper into the role of Wnt antagonism in cardiogenesis, we examined whether sFRP4 (which was overexpressed, as seen by quantitative RT-PCR studies) was activated by promoter demethylation. The gene structure of *Nkx2.5* and sequences of the promoter region are represented in Fig. 3a. After bisulfite conversion of the DNA from untreated MSCs (U) and differentiated cardiomyocytes (D), we amplified *Nkx2.5* promoter regions and sequenced the products (Fig. 3b). It was seen that after differentiation with DC301 + DC302, 6 out of the 10 CpG islands underwent demethylation in D (Fig. 3c). We could also see clearly that the unmethylated specific primer DNA product was increased in D while the methylated specific DNA product was high in U (Fig. 3d). Significantly, a remarkable change was observed in the *sFRP4* profile after cardiac differentiation. For the first time, we showed that a Wnt antagonist was activated in cardiogenic differentiation from MSCs by promoter demethylation. After alignment of the bisulfite sequences of U, D, and genomic DNA, we observed that 7 out of the 26 CpG islands in the *sFRP* promoter regions underwent demethylation in the cardiac differentiated cells (D) (Fig. 4b, c). The *sFRP4* promoter region and the sequences are shown in Fig. 4a. Confirmation of demethylation of CpG in differentiated cells was provided by the presence of unmethylated specific sFRP4 DNA in differentiated cells (Fig. 4d).

### In-vivo engraftment and reversal of cardiac damage by MSC-derived cardiomyocytes

#### Establishment of cardiac fibrosis in mice by treatment with doxorubicin

**Fig. 5:**
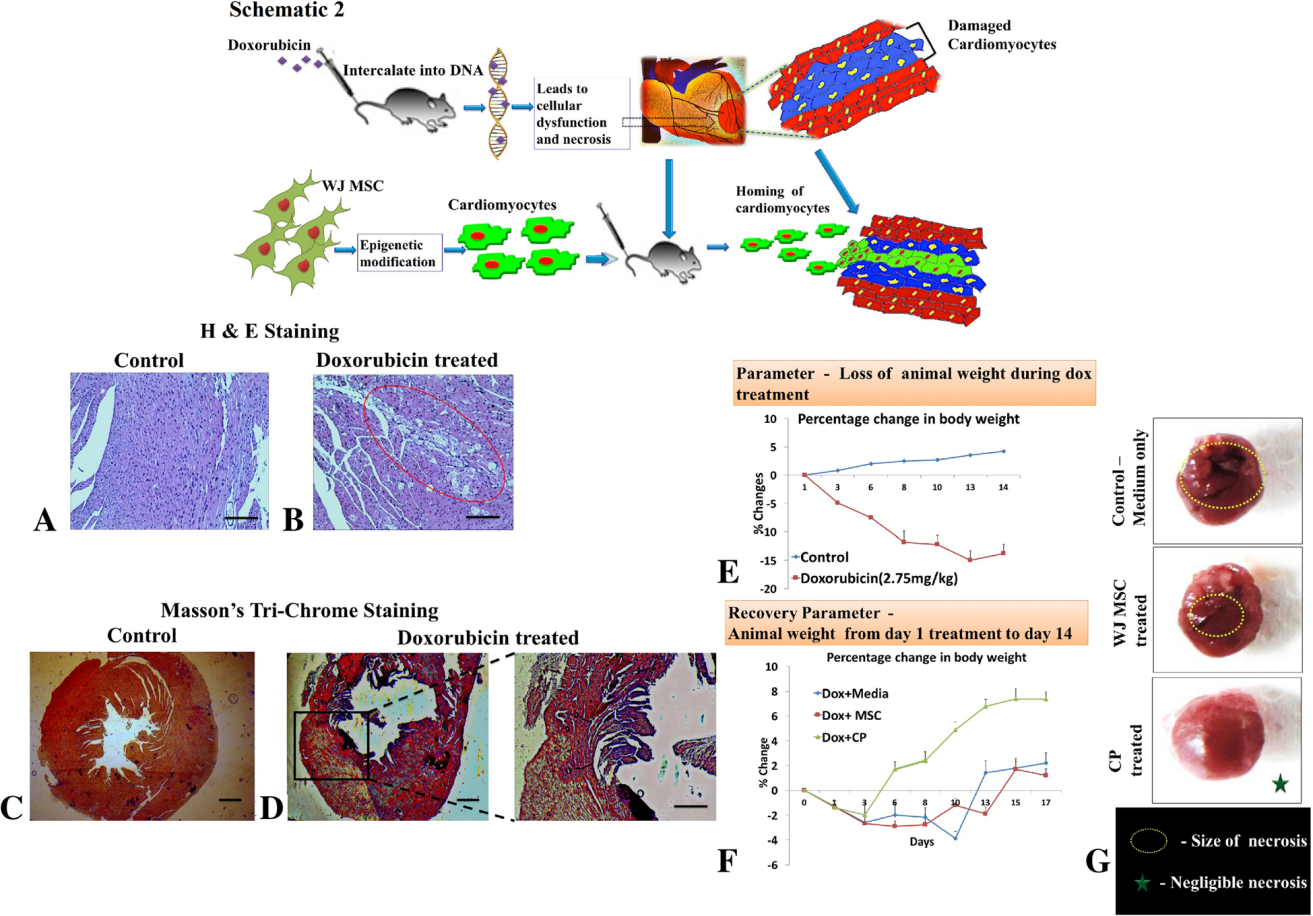
In vivo transplantation and engraftment of WJMSCs and CPs, and restoration of cardiac damage. *Schematic 2*: graphical representation of the induction of cardiotoxicity by doxorubicin in mouse and the engraftment of MSC-derived cardiomyocytes to restore the damaged cardiac tissue. **a–d** Induction of cardiac fibrosis in mice by treatment with doxorubicin. H&E images of (**a**) untreated mouse heart without fibrosis and (**b**) doxorubicin-treated (2.5 mg/kg body weight in six equal injections on alternative days) mouse heart indicating fibrotic regions as shown in the *red circle* (*scale bars* = 100 μm, *n* = 4). Masson’s trichrome staining of (**c**) control mouse heart (*scale bar*, 200 μm) and (**d**) doxorubicin-treated mouse heart showing the heart cross-section (*scale bar* = 200 μm (*left*) and *scale bar* = 50 μm (*right*)) indicating clearly increased fibrosis (*blue square*) in the cardiac muscle compared to the control group (*n* = 4). **e**–**g** Transplantation of WJMSCs and cardiac progenitors (*CP*) into mice induced with cardiac fibrosis. (**e**) Percentage change in body weight, showing a decrease in body weight after the doxorubicin (*dox*) treatment period. (**f**) Percentage change in body weight, indicating an increase in body weight in the mice transplanted with CP cells. (**g**) Representative image of a heart, indicating necrosis in plain medium-treated control, reduced necrotic area in mice transplanted with undifferentiated MSC, and negligible necrosis in CP-transplanted mice; necrotic regions indicated in *dashed circles. WJMSC* Wharton’s jelly mesenchymal stem cell, *H&E* hematoxylin and eosin (Color figure online)

We first established a cardiac injury model in C57BL/6 mice by inducing cardiotoxicity with a known cardiotoxic oncotherapeutic drug, doxorubicin. After injection of 2.75 mg doxorubicin/kg body weight in six equal injections on alternate days, we examined heart sections by H&E staining and observed fibrosis in dox-treated mice (Fig. 5b, indicated in the red circle) when compared to the control (Fig. 5a). Masson’s trichrome staining revealed increased cardiac fibrosis, as indicated by fibrosis-specific collagen staining (blue) (Fig. 5d) in comparison to medium-treated mice (Fig. 5c). The plan and rationale of the in-vivo studies for the induction of cardiotoxicity by doxorubicin and the engraftment of MSC-derived cardiomyocytes for cardiac restoration is depicted schematically (Fig. 5 Schematic 2).

#### Transplantation of WJMSCs and cardiac progenitors into mice induced with cardiac fibrosis

After inducing cardiac injury, mice were injected with WJMSCs or CP to study the reversal of cardiac damage, and were compared with mice that received only cell-free medium. Initially, after the dox treatment period, there was a decrease of about 15% in body weight (Fig. 5e). Body weight was measured every alternate day, post cellular injection. There was a 9% increase in body weight in CP-injected animals (Fig. 5f). After 18 days post injection, the animals were sacrificed and the necrotic areas of the heart studied. Necrotic areas were slightly reduced in mice that received WJMSCs (Fig. 5g) when compared to the control (Fig. 5g). Significantly, we observed that there was no necrosis in the mice receiving CP treatment (Fig. 5g).

**Fig. 6:**
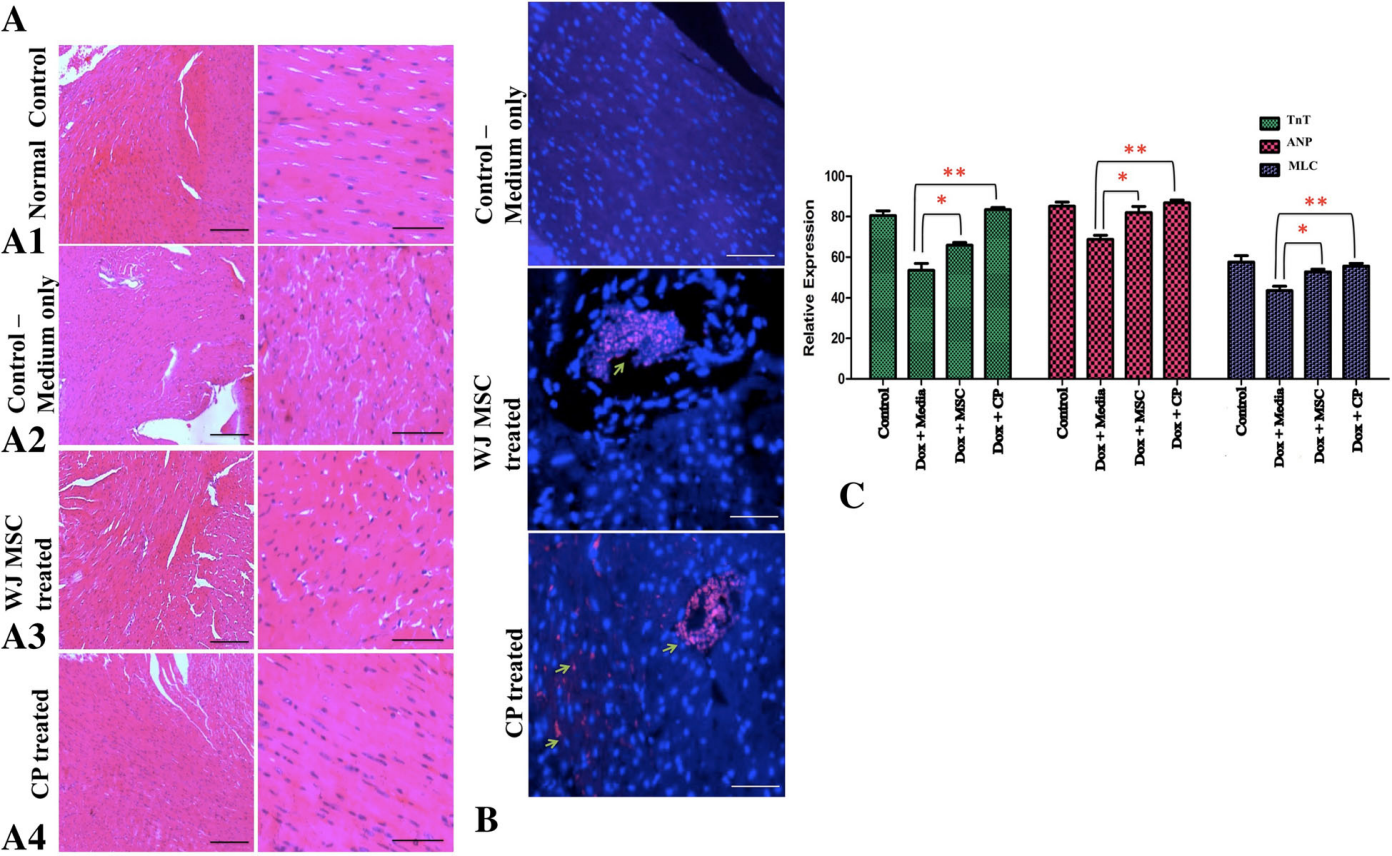
Staining of myocardial sections after MSC transplantation and engraftment. **a** Representative H & E staining of myocardial sections of normal heart (*A1*), control medium- treated mice showing necrotic regions at *scale bars*, 200μm (*left panel*) and *scale bars*, 100μm (*right panel*) (*A2*), MSC-transplanted mice showing reduction of necrotic regions at magnification of *scale bars*, 200μm (*left panel*) and *scale bars*, 100μm (*right panel*) (*A3*), and CP-transplanted mice showing loss of necrosis and restoration of normal cardiac tissue architecture (*scale bars*, 200μm (*left panel*) and *scale bars*, 100μm (*right panel*) (*A4*)). **b** Cell tracker-labeled MSCs to study homing of cells to the cardiac region. Control medium-treated mice showed lack of cell tracking labels (*top*). MSC-transplanted group showed clear homing of the cell tracker positive cells (indicated by *arrows*) (*middle*). Nuclei counterstained with DAPI (*blue*). CP-transplanted group indicated homing of cell tracker positive cells (indicated by *arrows*) (*bottom*) with significant decrease in the size of the cardiac damage. Nuclei counterstained with DAPI. **c** Analysis of cardiac genes in the necrotic regions of mice myocardium. Quantitative RT-PCR of *troponin T* (*TnT*), *atrial natriuretic peptide* (*ANP*), and *myosin light chain* (*MLC*) control untreated, medium-only-treated, MSC-transplanted, and CP-treated groups showed the recovery of *ANP* and *MLC* expression in MSC-treated and CP-treated groups following Doxorubicin treatment (*p < 0.05, **p < 0.01, *n* = 4)*. WJMSC* Wharton’s jelly mesenchymal stem cell, *H&E* hematoxylin and eosin, *CP* cardiac progenitors, *Dox* doxorubicin (Color figure online)

H&E staining showed that, when compared to the control medium (Fig. 6aA2), MSC-transplanted mice showed a marked reduction in necrotic areas (Fig. 6aA3). Remarkably, we could see a complete loss of necrosis in CP-treated mice (Fig. 6aA4), with tissue architecture similar to the untreated normal mouse heart (Fig. 6aA1).

### Engraftment of WJMSCs and cardiac progenitors, and formation of functional cardiomyocytes

To establish homing and engraftment of MSCs and CPs to the site of injury, we labeled cells with a red fluorescent cell tracker and found that mice injected with both MSCs and CPs showed fluorescence in the cardiac sections. In CP-treated mice, there was a visible decrease in the size of the fibrotic region and homing cluster (as indicated by the arrow at higher resolution) compared to MSC-treated mice (Fig. 6b). We established the gene expression of cardiac markers *ANP*, *MLC*, and *TnT* by quantitative RT-PCR. It was clearly seen that MSC-treated and CP-treated mice had higher expression of these genes compared to the Dox-treated mice, and CP-treated mice had the highest fold expression, showing nearly complete recovery of cardiac functional gene expression (Fig. 6c).

## Discussion

The regulation of expression of transcription factors during cardiogenesis is orchestrated by epigenetic changes that establish and maintain the expression of cardiac-specific genes. This study demonstrates the robust derivation of functional cardiomyocytes from WJMSCs using a combination of epigenetic modifiers. Major epigenetic reprogramming events associated with cardiogenesis are DNA methylation and histone acetylation [33]. A combination of inhibitors of DNA methylation and histone deacetylation has been used for the first time in our study to successfully derive cardiomyocytes from human WJMSCs. These chromatin remodelers initiated dynamic changes in the chromatin structure and possibly resulted in promoter accessibility of some genes, such as early stage cardiac transcription factors, *Nkx2.5* and *GATA4*, and the masking of other noncardiac and self-renewal genes.

Specification of these presumptive cardiogenic cells begins with the induction of the early transcription factor, Nkx2–5, regulated by Cerberus and possibly BMP2 [34, 35]. Nkx2–5 is a key protein guiding the mesoderm to become heart by initiating the synthesis of other cardiac transcription factors such as *GATA4* and *MEF2C*. These transcription factors, in turn, upregulate cardiomyocyte-specific proteins such as cardiac actin, atrial natriuretic factor, and alpha myosin heavy chain [36]. Early cardiac differentiation is marked by a change in the epigenetic landscape appropriate for cardiogenesis, including activation of cardiac-specific genes and repression of cell cycle progression genes and noncardiac genes [37]. Epigenetic modifications include covalent posttranslational modifications of either the DNA or the nucleosomes tightly regulated by specific enzymes; the main enzymes being DNA methyltransferases and histone deacetylases [38]. These enzymes block access to the promoter and histone regions, thereby grid-locking active transcription of the downstream genes. Suppression of *HDAC1* has shown to promote cardiogenesis by upregulating *Nkx2.5* [39]. Likewise, DNA demethylation of the CpG islands of the promoter regions of *Nkx2.5* and *GATA4* clears the path to access these promoters for active transcription. In our study, we used a combination of inhibitors of these enzymes and found that this efficiently generates cardiomyocytes. Therefore, our strategy has probably aided in an unlocking of the cardiac-specific promoters to drive active cardiomyogenesis. A schematic overview of the use of epigenetic modifiers for the development of cardiomyocytes from MSCs is represented in Fig. 2 (Schematic 1).

In the embryonic heart, Wnt signaling is one of the key modulators of cardiac development. The role of Wnt is also prominently evident upon pathological stress requiring adult heart remodeling. Blockage of Wnt signaling via Dkk has shown to significantly increase cardiac progenitors in embryonic stem cells [40]. Among the sFRPs studied, sFRP1 has a role in cardiac differentiation in *Xenopus* by promoting cardiac differentiation and regulating the normal heart size and cell fate within the cardiac mesoderm [41], and sFRP1-overexpressing mice showed reduced infarct size [42]. A positive role of canonical Wnt signaling has been reported in early cardiogenesis, followed by Wnt antagonism being more functional in later cardiac-specific gene expression [43]. In differentiated cardiomyocytes, we observed a clear effect of Wnt antagonism by an elevated expression of sFRP4 and a parallel increase of Dkk1 and Dkk3. Although the exact mechanism of activation is not yet known, we could observe epigenetic demethylation of sFRP4 promoter regions. One of the functions of sFRP4 overexpression would be to direct cells to exit the cell cycle and also inhibit proliferation of the differentiated cells. This would be similar to the effect of sFRP4 in glioma and head and neck cancers, where we demonstrated that it downregulates the proproliferation genes *cyclin D1* and *c-myc* [44, 45].

The Wnt noncanonical pathway, functioning independent of β-catenin, branches into the Wnt/JNK and Wnt/Ca^2+^ pathways, the latter route involving a release of intracellular calcium ions. This results in the activation of calcium-sensitive enzymes such as protein kinase C (PKC), calcium/calmodulin-dependent kinase (CaMK) II, or calcineurin. The noncanonical Wnt pathway is considered to inhibit canonical Wnt signaling [46], which could explain the expression of canonical Wnt antagonists and the activation of noncanonical Wnt/Ca^2+^ mediators seen in the present study. Expression of calcinuerin and its downstream mediators, such as the nuclear transcription factor CreB, indicates a clear initiation of the Wnt/Ca^2+^ axis. The accumulation of intracellular calcium in differentiated cardiomyocytes corroborates a definite involvement of the Wnt/Ca^2+^ pathway in cardiomyogenesis. Recently, a structural gene along the Wnt pathway, *vinculin*, has been reported to stave off aging of the heart [28]. *Vinculin* enhances the contractile function and hemodynamic stress tolerance of the heart by improving intercardiomyocyte connections and enhancing myofilament organization. The *vinculin* expressing differentiated cardiomyocytes obtained in our study could be an ideal cell type for cardiac muscle transplantation and restoration. Another gene that we found overexpressed in the differentiated cardimyocytes is *DDX20*, which belongs to the DEAD-box family of RNA helicases. *DDX20* assembles a corepressor complex with ETS, which functionally interacts with the E2F/pRB family of proteins inhibiting proliferation. This complex then directs a permanent cell cycle exit during terminal differentiation [47].

Nitric oxide is a pleiotropic signaling molecule acting as a vasoactive agent, neurotransmitter, and a free radical in mammalian systems. Accompanying cardiogenesis, we could clearly see a release of NO, which is the first time to our knowledge that NO release has been documented in cardiomyogenesis from human MSCs. NO activates class IIa HDACs and histone deacetylation in endothelial cells, and modulates mesodermal commitment in embryonic stem cells [48]. Exposure to NO donors enhanced the expression of Nkx2.5 and myosin light chain (MLC2) in human ESCs via cGMP signaling [49] The classic mode of action of NO is via the activation of guanylate cyclase [49]. Mujoo et al. reported that the expression of various subunits of soluble guanylyl cyclase (sGC alpha and beta 1 and 2) and nitric oxide synthase(s) (NOS-1, NOS-2, NOS-3) increased in cardiac differentiated cells compared to undifferentiated H-9 embryonic stem cells [49]. This pathway could be operational in our present model of cardiomyocyte differentiation from WJMSCs.

Next, we analyzed the gene promoter methylation status and found that not only *Nkx2.5* underwent promoter demethylation but also *sFRP4*, which revealed a demethylation at 7 of its 26 CpG islands, and could be one of the reasons for its activation. Lineage commitment genes in naïve stem cells are maintained in a repressed state by specific patterns of histone acetylation and methylation, and DNA promoter methylation at the CpG islands [50]. In the present study, induction of cardiac lineage differentiation by epigenetic modifiers was coupled by the activation of Wnt antagonists. This is the first report, to our knowledge, giving a glimpse into the role of promoter demethylation in sFRP activation. Furthermore, even partial demethylation of the sFRP4 promoter, as observed in this study, was sufficient to elicit the cardiogenic phenotype. Wnt inhibition is considered essential to allocate the cardiac progenitors into various cardiac derivatives [51]. sFRP1, along with Wnt8a, has been shown to be overexpressed in differentiating cardiomyocytes [52]. Silencing of sFRPs has been largely believed to be by promoter region hypermethylation, which represses their tumor-suppressive properties in glioma [53] and acute myeloid leukemia [54]. Epigenetic silencing of sFRPs, by which the tumor seeds are sown, could be the default status in undifferentiated stem cells. Relieving the epigenetic blockage could be the point in early development when crucial decisions of cell fate commitment are determined. Cardiac lineage commitment from undifferentiated stem cells could thus be favored by the CpG promoter demethylation of *sFRP4*, as observed in our study. MSCs showing promoter demethylation of *Nkx2.5* upon cardiomyogenesis has so far been reported only for cardiac MSCs [55]. These MSCs displayed changes at the promoter regions of early cardiac genes (including *Nkx2.5*) upon cardiac induction with an epigenetic cocktail. Our observation of CpG demethylation of the promoter region upstream of *Nkx2.5*, studied for the first time in the context of cardiac differentiation from WJMSCs, is in agreement with reports on cardiac MSCs and cardiac differentiation from ESCs [20].

Doxorubicin has long been known to induce cardiotoxicity and cause damage to cardiomyocytes by binding to topoisomerase 2b [56]. Given this effect of doxorubicin, this drug has been exploited for use as a cardiac damage-inducing drug in animal models [57], which we utilized in the present study. We found that cardiomyocytes differentiated in vitro were capable of restoration of cardiac tissue after doxorubicin-induced damage. Importantly, the cardiac progenitors were more efficient in repairing the damaged tissue, increasing the body weight and survival of the animals. There are several possible reasons to explain these findings. The progenitor cells can home easily to the site of injury, secrete paracrine factors, and can differentiate eventually into functional cardiomyocytes to improve cardiac function. Many reports have demonstrated that endogenous cardiac stem/progenitor cells or bone marrow-derived cells mobilize to the infarcted area after injury and recruit additional cells through a feedback mechanism [58]. It is also possible that the transplanted cardiac progenitor cells mobilize endogenous cardiac stem cells at the site of injury through paracrine secretions. This is in agreement with previous findings showing that 5aza treatment of BMMSCs (bone marrow mesenchymal stem cells) inhibited the ventricular scar from thinning and expanding, and minimized left ventricular chamber dilatation by improving the elasticity and contractility of BMMSCs [59]. Furthermore, the anti-fibrotic effect observed in the present study could be comparable to MSCs that stimulate the secretion of matrix metalloproteases and matrix metalloproteinase endogenous inhibitor, TIMP, by cardiac fibroblasts, as observed in a myocardial ischemic model in mice [60]. Overexpression of sFRP4 or Dkk1 by the cardiac primed cells could also function as a paracrine factor in normalizing cardiac function. A similar effect has been reported in MSCs overexpressing Akt or sFRP2, which inhibited adverse remodeling, fibrosis, and cardiomyocyte hypertrophy in a rat infarct model, suggesting a paracrine role for these secreted factors [61].

## Conclusion

We report here an efficient and robust differentiation of functional cardiomyocytes from Wharton’s jelly-derived MSCs using a cocktail of epigenetic modifiers, with an active involvement of Wnt mediators. These observations provide new insights into the involvement of Wnt regulators during cardiac differentiation. Priming of MSCs with an epigenetic cocktail may thus represent an efficient strategy for cardiomyocyte repopulation, which would be useful for cell-based therapy.

## Additional files

Additional file 1: Table S1. Presenting cardiac-specific gene primers, Table S2 presenting Wnt-related gene primers, Table S3 presenting stemness and noncardiac gene primers, and Table S4 presenting methylation-specific primers. (DOCX 20 kb)

Additional file 2: Figure S1. Showing characterization of WJMSCs. **A1** Photomicrograph of a confluent layer of MSCs obtained from Wharton’s jelly (*scale bar* = 200 μm, *n* = 3). **A2** Immunohistochemical staining of WJMSCs with vimentin and nuclei counter-stained with DAPI (*scale bar* = 100 μm, *n* = 3). **A3** Quantitative RT-PCR of MSC marker CD44 and pluripotency markers *Oct4*, *Nanog*, and *Sox2*, and negative CD34 mRNA expression of WJMSCs (*p < 0.05, **p < 0.01, *n* = 3). **B** Flow cytometric analysis of WJMSCs for MSC-positive CD markers CD73, CD90, and CD105, and negative marker CD34. **C1–C3** Trilineage differentiation of WJMSCs: Oil Red ‘O’ staining for adipocyte differentiation (**C1**), Von Kossa staining for osteocyte differentiation (**C2**), and Alcian Blue staining for chondrocyte differentiation of WJMSCs (**C3**) (*scale bar* = 100 μm, *n* = 3). (TIFF 9437 kb)

## Abbreviations

ANP: Atrial natriuretic peptide
CP: Cardiac progenitors
MSC: Mesenchymal stem cell
MSP: Methylation Specific Primers
TnI: Troponin I
TnT: Troponin T
WJMSC: Wharton’s jelly mesenchymal stem cell
