# Supplementary material for: Epigenetic reprogramming converts human Wharton’s jelly mesenchymal stem cells into functional cardiomyocytes by differential regulation of Wnt mediators
Source: Stem Cell Res Ther. 2017 Aug 14;8:185. doi: 10.1186/s13287-017-0638-7 (PMC5557557; doi:10.1186/s13287-017-0638-7)
Supplement: Supplementary file 1 — Presenting cardiac-specific gene primers, Table S2 presenting Wnt-related gene primers, Table S3 presenting stemness and noncardiac gene primers, and Table S4 presenting methylation-specific primers. (DOCX 20 kb) [file 13287_2017_638_MOESM1_ESM.docx]

**Additional file 1**

**TABLE S1 - Cardiac-specific gene primers**

| **GENE** | **PRIMERS** | **BASE PAIRS** | **ANNEALING(ºC)** |
| --- | --- | --- | --- |
| Cardiac actin | 5’GCCCTGGATTTTGAGAATGA 3’  5’GGCTGGAAGAGTGTCTCAGG 3’ | 133 | 60 |
| GATA4 | 5’ GATCTTCGCGACAGTTCCTC 3’  5’ CATGGCCAAGCTCTGATACA 3’ | 141 | 60 |
| Nkx2.5 | 5’ AAACTGCTCATCGCTCCTGT 3’  5’ TCTTTGACTGAGAAGGGCGT 3’ | 287 | 60 |
| MLC | 5’ TTGACAAGGAAGGAAATGGC 3’  5’ AATGTTATCCTCGTGCCCTG 3’ | 122 | 60 |
| TnT | 5’ ATGATGCATTTTGGGGGTTA 3’  5’ CAGCACCTTCCTCCTCTCAG 3’ | 116 | 60 |
| GAPDH | 5’ CAGAACATCATCCCTGCATCCACT 3’  5’ GTTGCTGTTGAAGTCACAGGCGAC 3’ | 181 | 60 |

**TABLE S2 – Wnt-related gene primers**

| **GENE** | **PRIMERS** | **BASE PAIRS** | **ANNEALING(ºC)** |
| --- | --- | --- | --- |
| Cre B | 5’ ATGACCATGGAATCTGGAGC 3’  5’ GGGCTAATGTGGCAATCTGT 3’ | 112 | 60 |
| Cal N | 5’ GATGATGGTGGGGAACAATC 3’  5’ GCCACCTACAACAGCACAGA 3’ | 127 | 60 |
| Vcl | 5’ GCCAAGCAGTGCACAGATAA 3’  5’ TCTTTCTAACCCAGCGCAGT 3’ | 279 | 55 |
| DDX20 | 5’ TGCCAGTAAACAGATGC 3’  5’ GTGCCAAAGGGTATGA 3’ | 166 | 60 |
| Dkk1 | 5’ TCCGAGGAGAAATTGAGGAA 3’  5’ CCTGAGGCACAGTCTGATGA 3’ | 157 | 60 |
| Dkk3 | 5’ CTGTGTGTCTGGGGTCACTG 3’  5’ GCTCTAGCTCCCAGGTGATG 3’ | 208 | 60 |
| sFRP1 | 5’ ATCTCTGTGCCAGCGAGTTT 3’  5’ AAGTGGTGGCTGAGGTTGTC 3’ | 202 | 55 |
| sFRP2 | 5’ AGGACAACGACCTTTGCATC 3’  5’ TTGCTCTTGGTCTCCAGGAT 3’ | 217 | 55 |
| sFRP3 | 5’ AAACTGTAGAGGGGCAAGCA 3’  5’ GGCAGCCAGAGCTGGTATAG 3’ | 227 | 55 |
| sFRP4 | 5’ CGATCGGTGCAAGTGTAAAA 3’  5’ GACTTGAGTTCGAGGGATGG 3’ | 181 | 54 |
| sFRP5 | 5’ GATGTGCTCCAGTGACTTTG 3’  5’ GCAGGGGTAGGAGAACATGA 3’ | 352 | 60 |

**TABLE S3 - Stemness and non-cardiac gene primers**

| **GENE** | **PRIMERS** | **BASE PAIRS** | **ANNEALING(ºC)** |
| --- | --- | --- | --- |
| CD44 | F- 5’ CATCTACCCCAGCAACCCTA 3’  R- 5’GGTTGTGTTTGCTCCACCTT 3’ | 271 | 60 |
| Sox2 | 5′ TCAGGAGTTGTCAAGGCAGAG 3′  5′ TCCGGGCTGTTTTTCTGGTT 3′ | 500 | 60 |
| Nanog | 5′ CCTCCTCCATGGATCTGCTTATTCA 3′  5′ GGAAAGGGACCGAGGAGTA 3′ | 265 | 58 |
| Oct4 | 5′AGGGCAAGCGATCAAGCA3′  5′GGAAAGGGACCGAGGAGTA3 | 166 | 58 |
| CD34 | 5’ GACCCTGATTGCACTGGTCA 3’  5’ GGTTCCAGCTCCAGCCTTT 3’ | 106 | 58 |
| Osterix | 5’ GGCACAAAGAAGCCGTACTC 3’  5’ CACTGGGCAGACAGTCAGAA 3’ | 218 | 60 |
| PPARγ | 5′ CCATGCTGTTATGGGTGAAA 3′  5′ TCAAAGGAGTGGGAGTGGTC 3′ | 276 | 60 |
| Collagen Type II | 5′ ATGATTCGCCTCGGGGCTCC 3′  5′ CATTACTCCCAACTGGGCGC 3′ | 576 | 60 |

**TABLE S4 – Methylation-specific primers**

| **GENE** | **PRIMERS** | **BASE**  **PAIRS** | **ANNEALING( ºC)** |
| --- | --- | --- | --- |
| sFRP4  Methyl | 5’ GGGTGATGTTATCGTTTTTGTATCGAC 3’  5’ CCTCCCCTAACGTAAACTCGAAACG 3’ | 113 | 55 |
| sFRP4 Unmethyl | 5’ GGGGGTGATGTTATTGTTTTTGTATTGAT 3’  5’ CACCTCCCCTAACATAAACTCAAAACA 3’ | 115 | 55 |
| NKX2.5 Methyl | 5’ ATTGTTTATCGTTTTTGTTATCGAG 3’  5’ CTAACAACTTCCCTACATAATACCG 3’ | 178 | 55 |
| NKX2.5 Unmethyl | 5’ TGTTTATTGTTTTTGTTATTGAGGT 3’  5’ AACAACTTCCCTACATAATACCACC 3’ | 174 | 57 |
| Bisulfite sFRP4 Promoter | 5’ GGTTGTAGTTGTTAAGGGAG 3’  5’ CAAAAAATTTCTTCCCCCA 3’ | 254 | 55 |
| Bisulfite NkX2.5 Promoter | 5’ GAGGTTTTTGGTTTAATGGTAGGTT 3’  5’ ACCCCTAACAACTTCCCTACATAATA 3’ | 169 | 56 |
