# Supplementary figures and images for: Epigenetic reprogramming converts human Wharton’s jelly mesenchymal stem cells into functional cardiomyocytes by differential regulation of Wnt mediators
Source: Stem Cell Res Ther. 2017 Aug 14;8:185. doi: 10.1186/s13287-017-0638-7 (PMC5557557; doi:10.1186/s13287-017-0638-7)

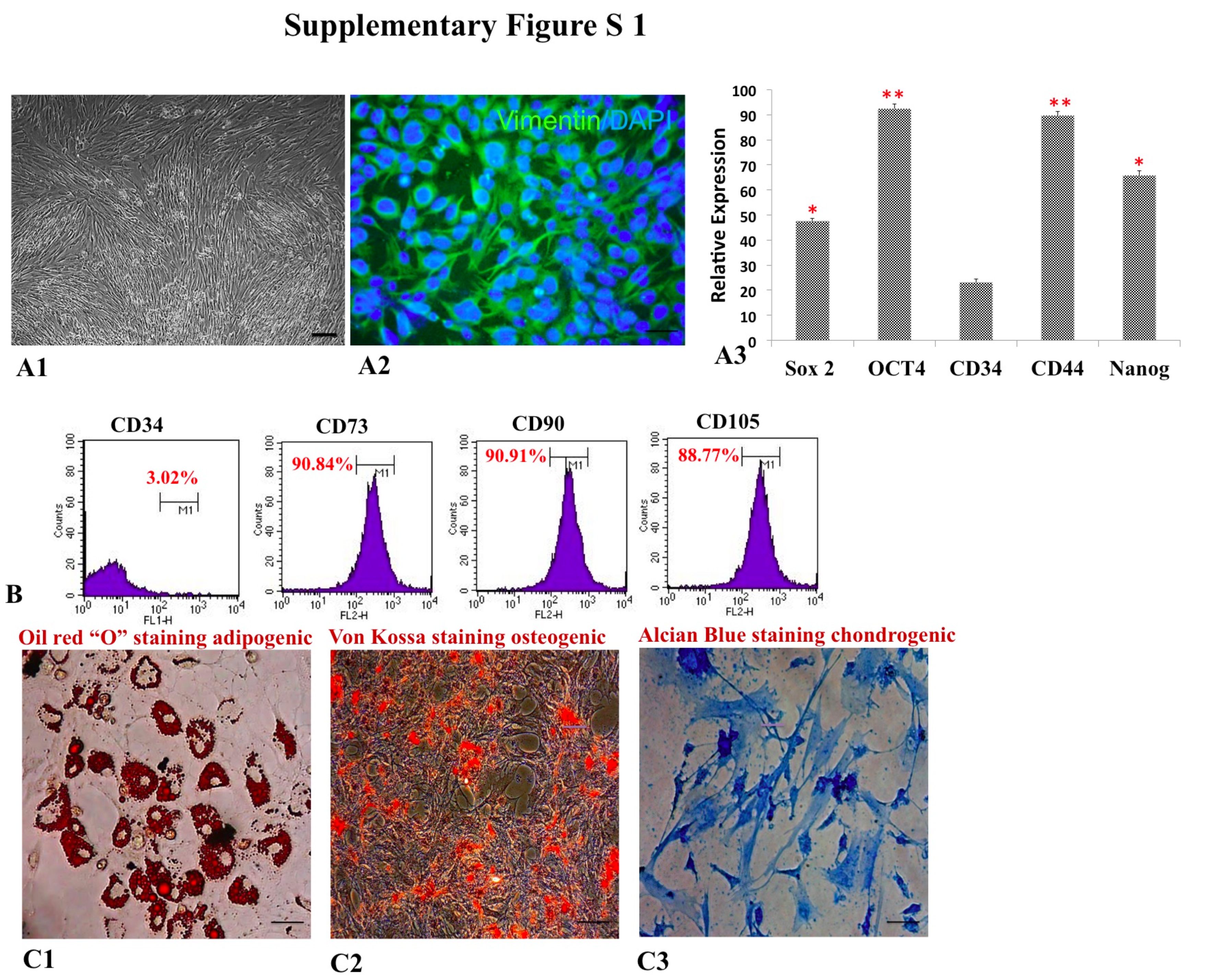

Supplement: Supplementary file 2 — Showing characterization of WJMSCs. A1 Photomicrograph of a confluent layer of MSCs obtained from Wharton’s jelly (scale bar = 200 μm, n = 3). A2 Immunohistochemical staining of WJMSCs with vimentin and nuclei counter-stained with DAPI (scale bar = 100 μm, n = 3). A3 Quantitative RT-PCR of MSC marker CD44 and pluripotency markers Oct4, Nanog, and Sox2, and negative CD34 mRNA expression of WJMSCs (*p < 0.05, **p < 0.01, n = 3). B Flow cytometric analysis of WJMSCs for MSC-positive CD markers CD73, CD90, and CD105, and negative marker CD34. C1–C3 Trilineage differentiation of WJMSCs: Oil Red ‘O’ staining for adipocyte differentiation (C1), Von Kossa staining for osteocyte differentiation (C2), and Alcian Blue staining for chondrocyte differentiation of WJMSCs (C3) (scale bar = 100 μm, n = 3). (TIFF 9437 kb) [file 13287_2017_638_MOESM2_ESM.tiff]
